# Distribution of *Kudoa thyrsites* (Cnidaria, Myxozoa) myoliquefactive stages in Northeast Atlantic mackerel (*Scomber scombrus*) inferred from qPCR and histology

**DOI:** 10.1007/s00436-022-07575-8

**Published:** 2022-06-18

**Authors:** Lucilla Giulietti, Heidi Johansen Nedberg, Egil Karlsbakk, Nachiket P. Marathe, Julia E. Storesund, Stig Mæhle, Ingrid Uglenes Fiksdal, Dawit Berhe Ghebretnsae, Arne Levsen

**Affiliations:** 1 Institute of Marine Research (IMR), Bergen, Norway; 2 Department of Biological Sciences (BIO), University of Bergen, Bergen, Norway

**Keywords:** *K. thyrsites*, Northeast Atlantic mackerel, Tissue distribution, ‘Soft flesh’, Myoliquefaction score categories, qPCR

## Abstract

*Kudoa thyrsites* is a myxosporean parasite (Cnidaria, Myxozoa) that infects the skeletal and cardiac muscle of Northeast Atlantic (NEA) mackerel (*Scomber scombrus*). Heavy infections are associated with *post-mortem* myoliquefaction of the host skeletal muscle which reduces the quality of the fish product. The biological infection characteristics of the parasite in NEA mackerel are poorly known. This study examined the distribution of *K. thyrsites* in various organs of NEA mackerel from the northern North Sea, and elucidates the relationship between density of infection, developmental stage and parasite distribution in the musculature, and the extent of visible flesh myoliquefaction. Quantitative polymerase chain reaction (qPCR) data showed that *K. thyrsites* is unevenly distributed in the somatic musculature of the fish host, with highest density in the anterior ventral muscle sections—the belly flaps. A weak positive correlation was observed between the level of myoliquefaction and the parasite density in the fish host muscle. This relationship was also reflected by the amount and distribution of parasite developmental stages seen during histological examinations. Histological findings indicate an association between the dispersion of free myxospores and the level of myoliquefaction of the fish host muscle. Visceral organs were also found infected using qPCR, although at lower densities compared to the musculature.

## Introduction

Among the members of the multivalvulid myxosporean genus *Kudoa*, there are at least 14 species that may cause *post-mortem* myoliquefaction of the fish host’s skeletal musculature, commonly known as the ‘soft flesh’, ‘jelly fish’ (*gelèfisk* in Norwegian), or ‘jellymeat’ condition. In this respect, one of the most important species is *K. thyrsites* which can cause ‘soft flesh’ in highly diverse teleost fish species worldwide. Some of those are among the economically most valuable fish species, including (farmed) Atlantic salmon (*Salmo salar*), coho salmon (*Onchorhynchus kisutch*), snoek (*Thyrsites atun* — the parasite’s type host), and Atlantic mackerel (*Scomber scombrus*). This condition is primarily caused by *post-mortem* digestive action of an enzyme, cathepsin L peptidase, released by the parasite into the surrounding somatic muscle tissue (Funk et al. 2008). Severely liquefied fish or fillets may be discarded due to poor condition at the harvest and processing stage of the supply chain, resulting in loss of industry revenue and unnecessary losses of food along the supply chain (Bolin et al. 2021). In addition, ‘soft flesh’-affected fish may reach consumers, resulting in loss of consumer confidence, and a potential reduction in the market value of products derived from wild or farmed fish stocks that are prone to *Kudoa*-infections.

The infection pattern, development, and *post-mortem* myoliquefactive property of *K. thyrsites* in cultured Atlantic salmon have been studied in Pacific North America (Whitaker and Kent 1991; Moran et al. 1999a; Marshall et al. 2015). Studies include the relationship between parasite density and muscle firmness (St-Hilaire et al. 1997a) or flesh quality (Dawson-Coates et al. 2003), as well as a quantitative assessment of *K. thyrsites* infection levels by applying quantitative polymerase chain reaction (qPCR) (Funk et al. 2007). However, an understanding of the possible relationship between the presence and distribution of *K. thyrsites* stages in fish muscle, and the extent of myoliquefaction, is still lacking. Additionally, various studies using a combination of different techniques (PCR based and/or histology, in situ hybridization ‘ISH’ and immunohistochemistry ‘ICH’) detected the parasite also in the cardiac muscle of Atlantic salmon (Moran et al. 1999b; Young and Jones 2005; Di Cicco et al. 2017).

Ware et al. (2014) demonstrated a nonuniform plasmodial distribution of *K. inornata* in the musculature of spotted seatrout (*Cynoscion nebulosus*) from South Carolina, USA. They found that the density of plasmodia in the anterior hypaxial muscle area was higher than in all other muscle areas examined, and independent of fish host size or age. Mature plasmodia of *Kudoa* species are filled with myxospores that may be released from ruptured plasmodia. It has been observed that myoliquefaction in Chinese sea bass (*Lateolabrax* sp.) infected with *K. lateolabracis* is associated with dispersion of spores (Yokoyama et al. 2004). In myoliquefied lumpfish (*Cyclopterus lumpus*) infected with *K. islandica*, the level of muscle degradation seems to be related to the number and dispersion of free mature myxospores (Kristmundsson and Freeman 2014).

To date, only little is known about the general infection biology of *K. thyrsites* in Northeast Atlantic (NEA) mackerel. In fact, there are only a few studies that describe the occurrence and myoliquefactive effect of *K. thyrsites* in NEA mackerel (Levsen et al. 2008; Giulietti et al. 2022; Højgaard et al. 2022), although the fish species is among the commercially most important fish stocks in the NE Atlantic. For example, Norwegian mackerel catches reached 212,000 metric tonnes in 2020 representing a value of catch of approximately 2.8 billion Norwegian krone (Economic and biological figures from Norwegian fisheries 2020, retrieved on 1st January 2022 from: https://www.fiskeridir.no/Yrkesfiske/Tall-og-analyse/Statistiske-publikasjoner/Noekkeltall-for-de-norske-fiskeriene). The NEA mackerel stock has recently undergone substantial changes in geographic distribution and feeding migration pattern, especially concerning the stock component that undertakes extensive migrations each year into the North and Norwegian Seas and beyond, including the southwestern parts of the Barents Sea and the waters around Iceland (Nøttestad et al. 2016; Olafsdottir et al. 2019). Recent findings by us suggest that the occurrence of *K. thyrsites*-induced ‘soft flesh’ in NEA mackerel caught in the northern North Sea has increased significantly over just a few years, typically ranging 0.8–1.5% between 2007 and 2018 while showing 2.0–3.7% occurrence in 2019 and 2020, respectively (Giulietti et al. 2022). This increase coincided with observations by the mackerel processing industry, of an apparent increasing proportion of ‘soft’ mackerel in recent years. This phenomenon can lead to serious economic losses for the pelagic fisheries industry due to reduced value of catches and even loss of reputation in important export destinations related to the risk of marketing inferior quality products. Thus, there is a need to increase the knowledge on the development and distribution of myoliquefactive stages of *K. thyrsites* in NEA mackerel, which would also be valuable information for the pelagic fisheries industry, in order to easily detect and discard ‘soft flesh’ affected mackerel before further processing and market release.

The aim of the present study was to examine the presence, density and distribution of *K. thyrsites* in various organs of NEA mackerel by qPCR, and to check for any coherence with the occurrence and distribution of parasite plasmodia and myxospores as assessed by histology and visual appearance of the fish flesh.

## Materials and methods

### Fish and sample collection

A total of 1400 Atlantic mackerel were sampled in October 2019 during a research cruise (IMR cruise no. 2019853) in the northern North Sea at approximately N58°18.22′ W00°37.55′. Prior to examination, total length (TL; cm) and total weight (TW; g) of each fish were recorded. Fish were then cool-stored (10 °C) on board and examined for *post-mortem* myoliquefaction at 6, 12, 18, 24, 36, and 48 h post catch. The musculature of all mackerel was examined by manual muscle texture testing and visual inspection of the muscle appearance (Levsen et al. 2008), i.e., whether the basic segmental myomere structure was intact or not. For preliminary microscopic analysis on board, two subsamples of muscle tissue were taken from each fillet side of fish showing signs of generalized or focally restricted ‘soft flesh’. Fresh muscle squash preparations were examined in a brightfield microscope for the presence of *Kudoa*-like myxospores (400 × magnification) following the procedures provided by St-Hilaire et al. (1997b). If spores were detected, then the fish was selected for further analyses. In total, 30 out of 57 ‘soft flesh’-affected mackerel, and 33 out of 1400 intact mackerel (negative control reference, NC) were randomly selected and divided into two groups (see below). During dissection of the fish, all muscle tissue samples were taken from the right fillet side leaving the left fillet for visual evaluation including liquefaction score ranking (for details, see below).

**Fig. 1:**
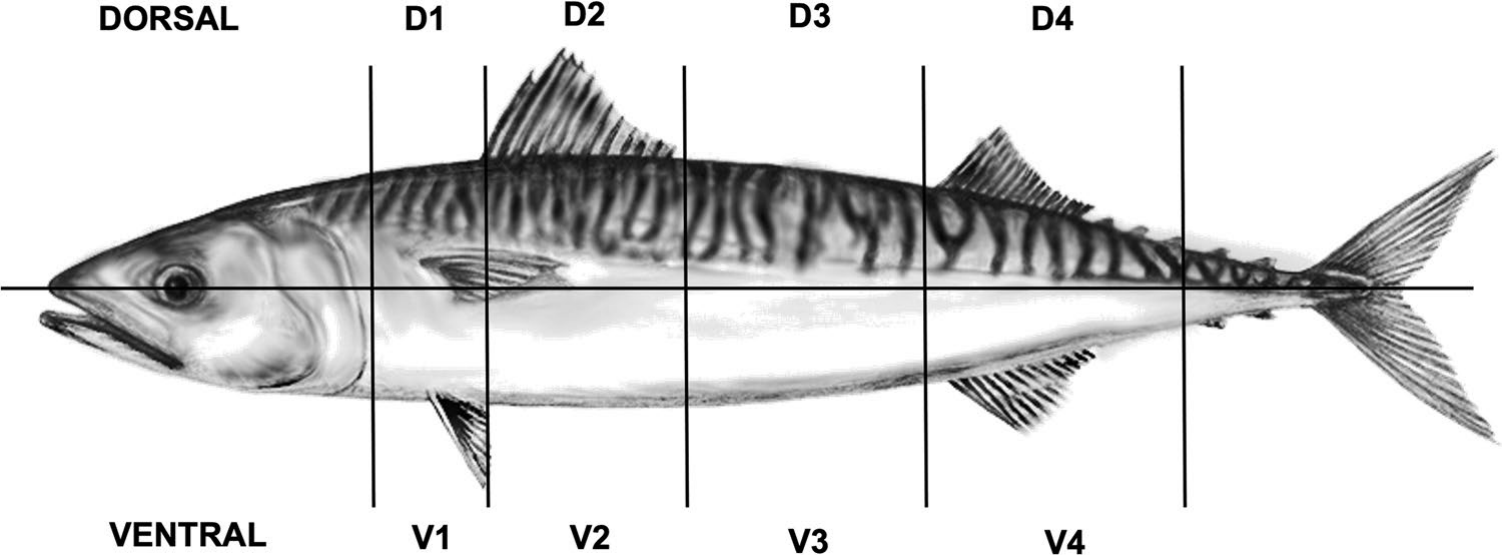
The eight skeletal muscle sections of Atlantic mackerel (left fillet) from which the tissue samples were collected (qPCR and histology). Each mackerel was divided into eight sections with four per dorsal (D) and ventral (V) fillet parts

Group 1. Tissue sampling for *Kudoa* sp. quantification (qPCR). Group 1 included 15 ‘soft flesh’-affected fish and 18 intact fish (negative control reference, NC) ranging 300–710 g in weight. These fish were used to examine the parasite density in the flesh and various visceral organs in terms of number of copies of the targeted small subunit ribosomal RNA gene (SSU rDNA) per mg of fish tissue (DNA molecules/mg) (qPCR). Tissue samples were dissected from skeletal muscle, kidney, spleen, liver, and heart (ventricle) using a scalpel and cut into samples of standardize size (1 cm × 1 cm × 1 cm). It was not possible to obtain blood samples from the fish. Two samples of standardized size (approximately 1 cm^3^) were collected from each organ in intact and ‘soft flesh’-affected fish. Skeletal muscle samples of approximately 1 cm^3^ were taken from 8 sites (Fig. 1), and each sample was stored in individual Eppendorf tubes at − 20 °C before DNA extraction.

Group 2. Tissue sampling for histological examination of *Kudoa* sp.-infected muscle tissue. Group 2 included 15 ‘soft flesh’-affected fish and 15 intact fish (negative control reference, NC) ranging in size 388–672 g. Six out of 15 fish were used for histological examination of skeletal muscle tissue in connection with the respective liquefaction score (see next section). Muscle blocks of standardized size (approximately 1 cm^3^) were collected from 8 tissue sites (see previous section) (Fig. 1), fixed in 4% buffered formaldehyde solution (pH 6.9) (Merck KGaA, Darmstadt, Germany) and transferred to 70% ethanol after 48 h. One fish (out of 15) showing particularly liquified fillets (liquefaction score 5) could not be processed for histological examination.

### Visual assessment and myoliquefaction scoring

**Fig. 2:**
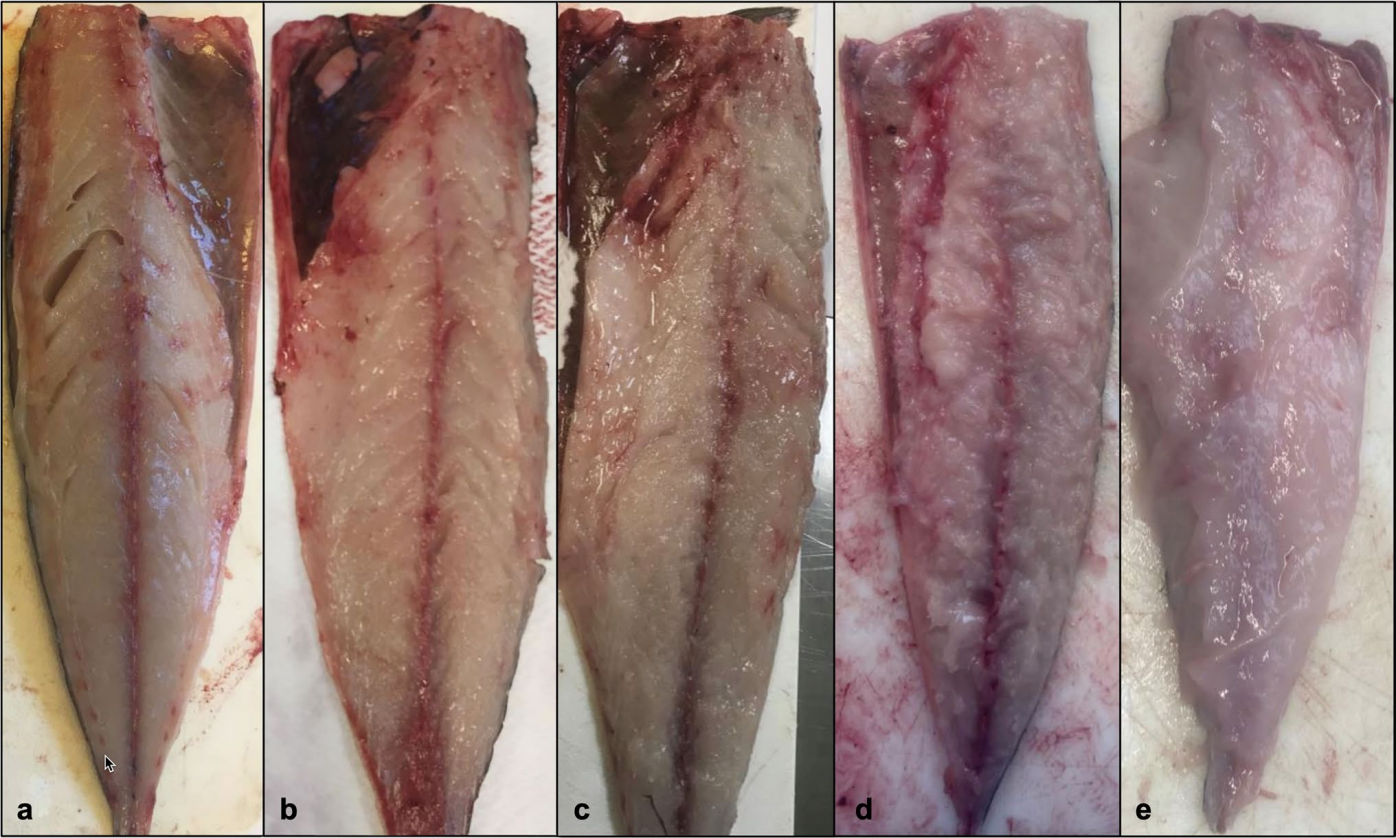
Examples of myoliquefaction scores 1–5 in fresh mackerel fillets after 48 h of cool storing. **a** Score 1; **b** Score 2; **c** Score 3; **d** Score 4; **e** Score 5

Left fillets of fish belonging to Groups 1 and 2 were subjected to visual evaluation of flesh degradation, including liquefaction scoring. The visual evaluation categories proposed by Funk et al. (2007) for Atlantic salmon were modified, to better reflect the *Kudoa*-induced muscle liquefaction conditions in mackerel. The score system has five categories (Fig. 2). These are 1 (normal) = intact muscle tissue; 2 (slight) = basic myomere structure largely intact but with some minor foci showing slight myomere deterioration and some loss of firmness; 3 (moderate) = larger areas showing deteriorated myomere structure with only smaller intact areas but still with some overall firmness; 4 (strong) = only very few focally restricted and barely intact myomere areas and almost complete loss of firmness; 5 (severe) = myomere structure completely destroyed and muscle tissue without any firmness, showing viscous consistency throughout. Differences in muscle degradation score among fillet sections were also noted.

### DNA extraction

Tissue samples of fish belonging to Group 1 (skeletal muscle, kidney, spleen, liver, and heart ventricle) were individually homogenized by TissueLyser (Qiagen, Hilden, Germany), in sterile PBS buffer with four glass beads. Genomic DNA extraction was performed on 60 mg of the homogenate using DNeasy® Blood and Tissue Kit (Qiagen, Hilden, Germany) and following the manufacturer’s instructions. DNA concentration was determined using Qubit™ 2.0 Fluorometer (Life Technologies, Thermo Fisher Scientific, Waltham, MA, USA) and normalized to 10.0 ng/μl.

### qPCR analyses

The qPCR for *K. thyrsites* was conducted using the assay designed to amplify a part of the SSU rDNA of the parasite (target, 82 bp product) (Funk et al. 2007). The cytochrome *B* (*cyt B*) of Atlantic mackerel was used as endogenous control (reference, 60 bp product) (Velasco et al. 2013). The analyses were performed following the procedures as detailed by Giulietti et al. (2022). DNA extracted from infected and uninfected mackerel tissues previously examined (Giulietti et al. 2022) were used as positive and negative controls, respectively. Negative and positive controls were included for each set of amplification. *K. thyrsites* density in mackerel was measured as SSU rDNA copies per mg of fish tissue (DNA molecules/mg). The calculation was carried out as follows: [(number of copies of SSU rDNA) × (35 μl of total elution volume of extracted DNA)/20 ng qPCR DNA input] mg − 1 of fish tissue.

### Molecular identification

*K. thyrsites* qPCR-positive samples were randomly selected among infected fish for PCR amplification and sequencing, in order to confirm amplicon identity. The SSU rDNA of 35 samples representing both muscle (*N* = 20) and visceral organs (*N* = 15) was amplified by PCR and sequenced (Sanger) following the procedure reported in Giulietti et al. (2019).

### Histological analyses

Skeletal muscle samples of fish belonging to Group 2 were embedded in paraffin, sectioned at 3 μm using standard histological techniques and stained with Giemsa or hematoxylin erythrosine saffron (HES). The tissue sections were scanned with NanoZoomer S60 (Hamamatsu Photonics Europe, Ammersee, Germany) and visualized at 40 × magnification with NDP view2 software (Hamamatsu Photonics). The resulting images were examined for histological changes and level of degradation of the muscle tissue in relation to the assigned liquefaction score of actual fish, and how the *K. thyrsites* plasmodia or spores were distributed within the musculature.

### Statistical analyses

All statistical analyses were performed using Statistica® 13.3.0 (TIBCO Software Inc., CA, USA). The data were non-normal (Shapiro–Wilk test and Levene’s tests), so rank-based nonparametric tests were used. The level of statistical significance was set to 95% (*p* < 0.05).

## Results

### qPCR analyses

**Fig. 3:**
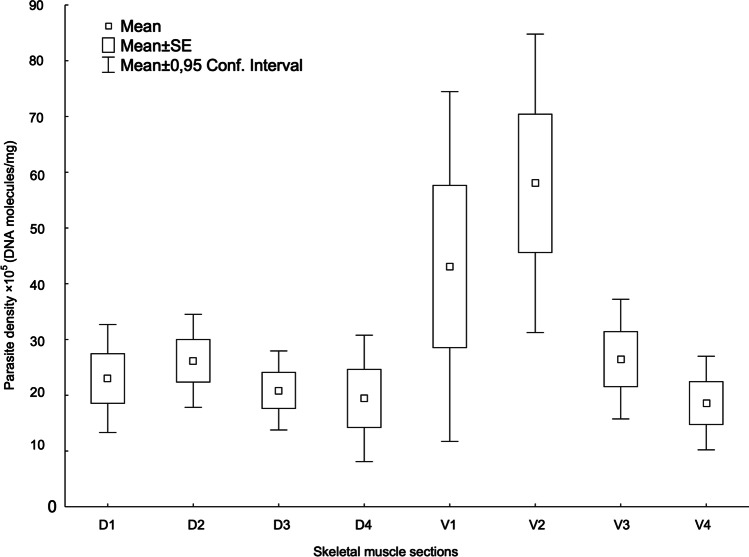
Density of *K. thyrsites*, expressed as mean (± SE ± CI) DNA molecules/mg × 10^5^, in 8 sections of the right fillet side’s musculature in *n* = 15 ‘soft flesh’-affected Atlantic mackerel. For legend of muscle section division (see Fig. 1)

*K. thyrsites* density (DNA molecules/mg) varied significantly among the skeletal muscle sections of the mackerel (Friedman ANOVA, *χ*^2^ = 25.4, *p* < 0.001). Across the 15 hosts, the V1 and V2 muscle sections contained 18 and 25% (totally 43%) of the parasite gene copies recorded. The density in V2 was significantly higher compared to all other sections except of V1 (Wilcoxon matched pairs test, *p* < 0.05). Parasite density in V1 differed only from the posterior dorsal and ventral muscle sections D3, D4, and V4 (Wilcoxon matched pairs test, *p* < 0.05) (Fig. 3; Table 1). The two smallest fish among the ‘soft flesh’–affected mackerel of Group 1 (weighing 388 and 410 g), accounted alone for 42% of all parasite target gene copies recorded in sections V1 and V2 of the sample.

**Table 1:** Occurrence and density (DNA copies/mg) of *Kudoa thyrsites* in skeletal muscle (8 sections) and visceral organs of Atlantic mackerel assessed by qPCR

		‘Soft flesh’-affected fish (*N* = 15)		Intact fish (*N* = 18)	
		Overall *P* = 100% (*n* = 15/15)		Overall *P* = 89% (*n* = 16/18)	
	Tissue sites	Occurrence *Kudoa*-pos. samples (%)	Density (mean ± SD (min–max))	Occurrence *Kudoa*-pos. samples (%)	Density (mean ± SD (min–max))
Skeletal muscle	D1	15 (100)	23 × 10^5^ ± 17 × 10^5^ (1.8 × 10^5^–57 × 10^5^)	-	-
	D2	15 (100)	26 × 10^5^ ± 15 × 10^5^ (7.0 × 10^5^–62 × 10^5^)	4 (22)	3.0 × 10^−1^ ± 6.5 × 10^−1^ (0–2.0)
	D3	15(100)	20 × 10^5^ ± 13 × 10^5^ (2.7 × 10^5^–44 × 10^5^)	-	-
	D4	15 (100)	19 × 10^5^ ± 20 × 10^5^ (3.6 × 10^5^–86 × 10^5^)	-	-
	V1	15 (100)	43 × 10^5^ ± 57 × 10^5^ (4.4 × 10^5^–23 × 10^5^)	-	-
	V2	15 (100)	58 × 10^5^ ± 48 × 10^5^ (4.3 × 10^5^–15 × 10^5^)	-	-
	V3	15 (100)	26 × 10^5^ ± 19 × 10^5^ (5.7 × 10^5^–74 × 10^5^)	-	-
	V4	15 (100)	18 × 10^5^ ± 15 × 10^5^ (5.0 × 10^5^–62 × 10^5^)	-	-
Visceral organs	Heart	13 (87)	6.4 × 10^2^ ± 14 × 10^2^ (0–48 × 10^2^)	5 (28)	5.1 ± 15 (0–65)
	Spleen	4 (27)	2.4 × 10^2^ ± 8.2 × 10^2^ (0–32 × 10^2^)	1 (6)	28
	Liver	4 (27)	2.7 × 10^2^ ± 9.4 × 10^2^ (0–36 × 10^2^)	10 (56)	43 ± 1.7 × 10^2^ (0–7.3 × 10^2^)
	Kidney	12 (80)	5.0 × 10^2^ ± 9.0 × 10^2^ (0–35 × 10^2^)	4 (22)	48 ± 95 (0–2.9)

**Fig. 4:**
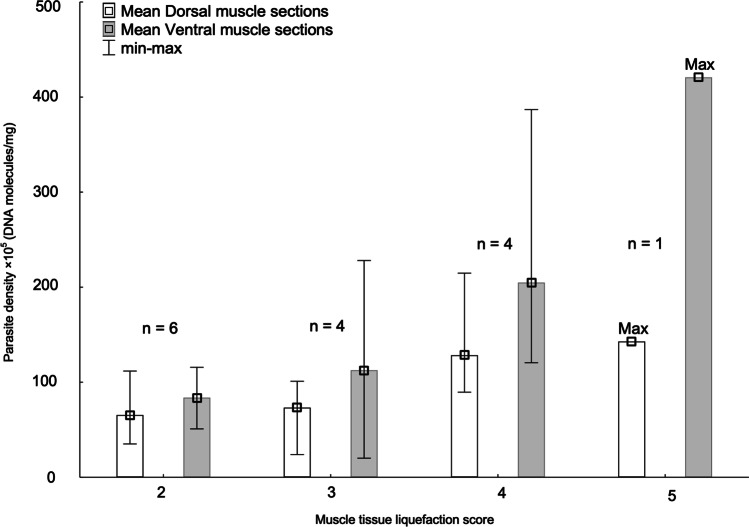
Density of *K. thyrsites*, expressed as mean (min–max) DNA molecules/mg × 10^5^, per muscle tissue liquefaction score in the dorsal and ventral sections of the right fillet side’s musculature in *n* = 15 ‘soft flesh’-affected Atlantic mackerel

The two smallest fish showed highest density, and were also those with highest muscle tissue liquefaction score, i.e., 4 and 5, respectively, thus contributing considerably to the significant trend of increasing myoliquefaction score with increasing parasite density in the ventral muscle sections (Spearman’ s rank test, *r*^2^ = 0.55, *p* < 0.002) (Fig. 4). However, there was no significant effect of fish body size (weight) on neither parasite density nor liquefaction score in either of the main muscle sections (dorsal or ventral). The hypaxial ventral muscle tissue sections V1–V2 that largely comprise the belly flaps tended to be thinner than the corresponding epaxial sections (D1–D2) (see Fig. 1), and it was not possible to visually assess any differences in muscle degradation between these fillet sections in ‘soft flesh’-affected mackerel showing liquefaction scores 3, 4, or 5. D2 was the section where the first signs of ‘soft flesh’ usually occurred during texture testing in all fish examined.

In addition to the somatic musculature, the parasite was detected by qPCR in the heart ventricle, spleen, liver, and kidney samples, although at lower level compared to the flesh (Table 1). Among these tissues, both parasite prevalence and density were highest in the heart, 87% and up to 4819 DNA molecules/mg, respectively. Similar to the trend in the musculature, the smallest of the ‘soft flesh’-affected mackerel (388 g) accounted for most of the parasite density ranging from 47% in the kidney to 90% in the liver of all parasite target gene copies recorded in the four internal organs screened within the actual fish sample.

### Molecular identification

*Kudoa* SSU rDNA sequences (1200 bp in length) were obtained from 20 randomly selected muscle samples and 15 visceral organ samples. All sequences were identical and matched 100% with a SSU rDNA sequence of *K. thyrsites* from mackerel caught in the North Sea (GenBank accession no. EU154349).

### Histological assessment

Histological examination revealed no evidence for inflammatory host responses in or between muscle fibres of mackerel that contained developing or mature *K. thyrsites* plasmodia. This observation was independent of the liquefaction score assigned to the actual fish fillets.

**Fig. 5:**
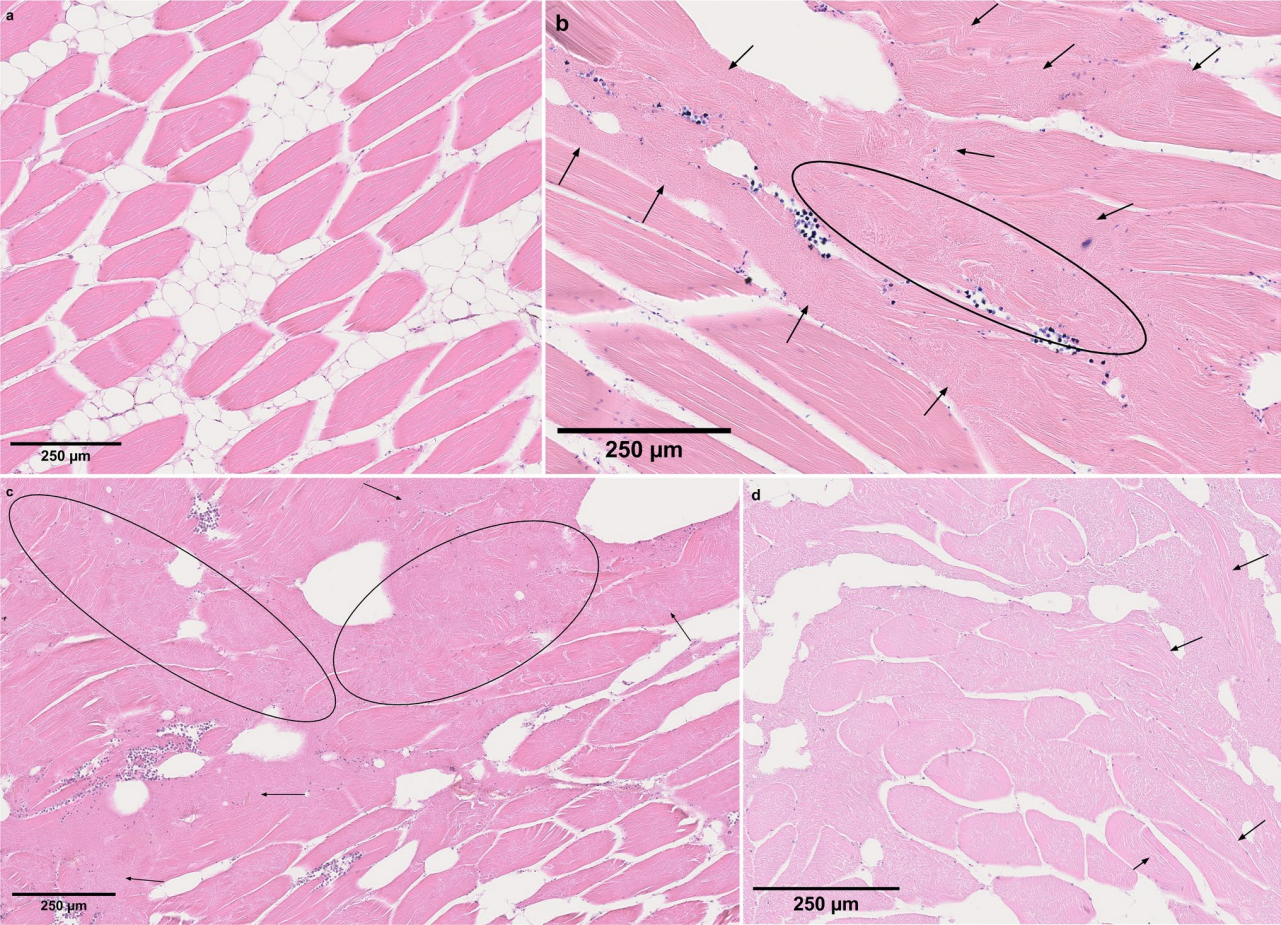
Histological sections of somatic musculature of Atlantic mackerel (HES or Giemsa). **a** Score 1 (uninfected) with intact muscle fibres; **b**, **c** *Kudoa thyrsites* infected soft fillet with liquefaction scores 2 and 3, respectively. Histological sections showed mainly intact muscle fibres with some focally restricted degraded areas (ellipses and arrows), which are larger and more pronounced in score 3 fillet. **d** *Kudoa thyrsites* infected soft fillet with liquefaction scores 4 showing only a few scattered barely intact muscle fibres (arrows) between the liquefied tissue areas

Histological observations showed that the areas macroscopically considered liquefied had confluent muscle fibres with disorganized, fragmented or lost striation (Fig. 5b, c, d). Histological differences in the appearance of affected muscle tissue were present between fillets assigned liquefaction score 2 and 3, and those scored 4. While the muscle fibres of score 2 and 3 fillets were mostly intact and had only focally liquefied areas (Fig. 5b, c), the sections of score 4 fillets tended to show multifocal or even generalized liquefaction, with only a few scattered intact muscle fibres between the liquefied tissue areas (Fig. 5d). The same general pattern was observed in sections of both dorsal and ventral fillet samples.

**Fig. 6:**
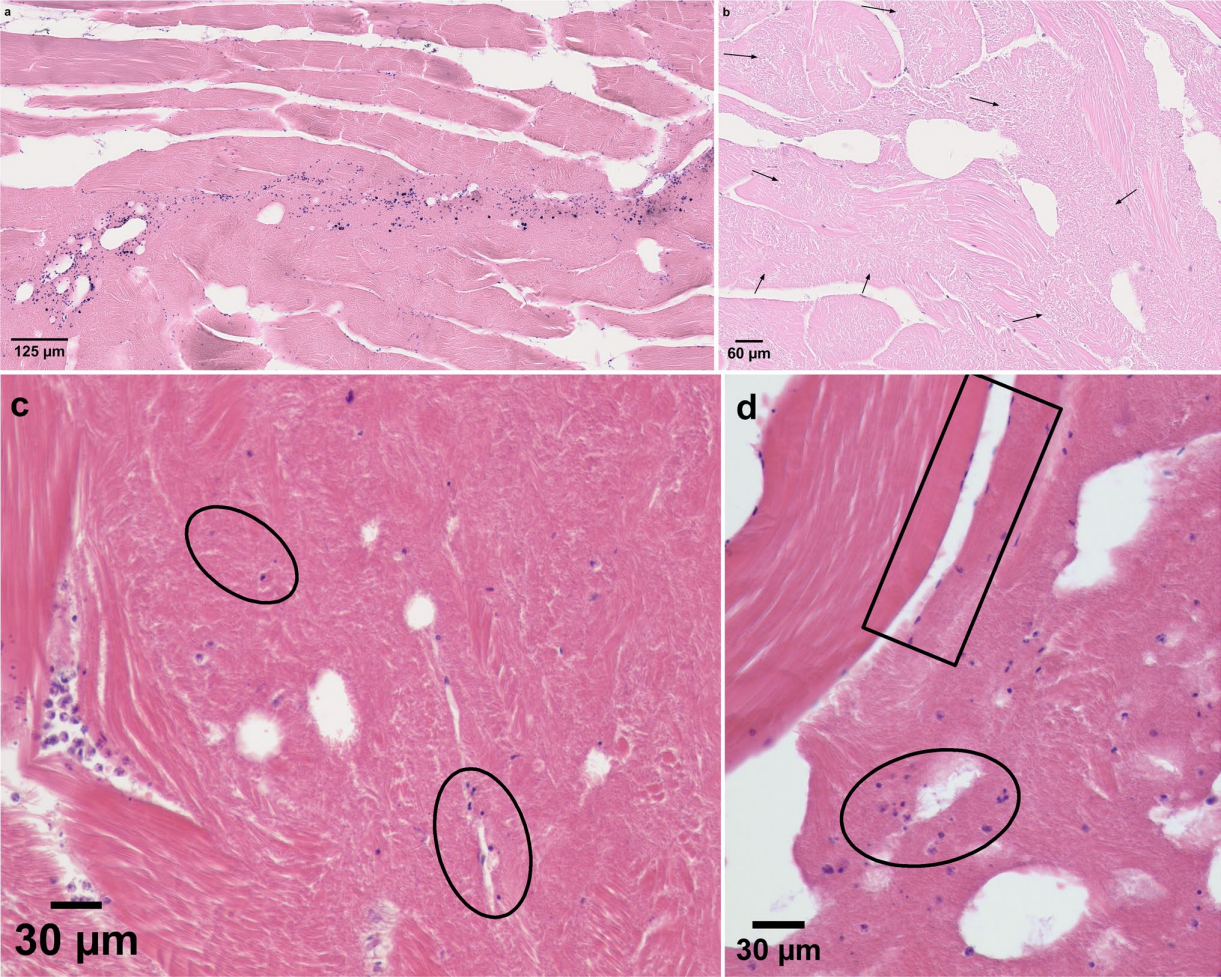
Histological section of *K. thyrsites* in somatic musculature of Atlantic mackerel (Giemsa). **a** Score 2 fillet, showing degraded muscle tissue closely associated with free myxospores (i.e., not confined to any plasmodia); **b** Score 4 fillet, with degraded areas with few or none myxospores (arrows); **c** Score 4 fillet, with deeply stained spots (ellipses) scattered within strongly degraded tissue areas, in the vicinity of aggregations of free myxospores; **d** Spores and deeply stained spots scattered within strongly liquefied tissue areas (ellipse). The spores show different appearance compared to myofibre nuclei in the close vicinity (rectangle) (score 4 fillet)

Mature *K. thyrsites* spores still confined within intact plasmodia were usually not associated with any liquefaction of the surrounding muscle fibres. Liquefactive foci were associated with the presence of dispersed myxospores (Fig. 6a). This was most pronounced in fillets assigned liquefaction score 2 and 3 which had numerous free myxospores, i.e., not confined to any plasmodia, within or in close proximity to clearly liquefied tissue areas (Fig. 6a). In score 4 fillets, free *K. thyrsites* spores were less numerous or even absent in clearly liquefied muscle tissue (Fig. 6b). In the sections of liquefaction scores 2–4, but especially in the score 4 sections, deeply stained spots were frequently observed. These spots were scattered within strongly liquefied tissue areas, and sometimes in the vicinity of aggregations of free myxospores (Fig. 6c). In some cases, spores and deeply stained spots scattered within strongly liquefied tissue areas seemed about to be dissolved (Fig. 6d).

## Discussion

The present study is the first to quantitatively assess the occurrence and density of the myxosporean parasite *K. thyrsites* in the somatic musculature and visceral organs of NEA mackerel. *K. thyrsites* was unevenly distributed in the somatic musculature, with clearly higher density in the anterior ventral muscle areas (V1 and V2), which correspond to the anterior part of the belly flap. Moreover, there was a significant trend towards higher myoliquefaction score with increasing parasite density in the ventral muscle sections (V1–V4).

An uneven distribution of *K. thyrsites* has also been reported from farmed Atlantic salmon by Dawson-Coates et al. (2003), who found a large variability in both plasmodia and spore numbers among individual fish and among muscle samples within a fish. Similarly, Funk et al. (2007) observed a significant intra-fish variability in Atlantic Salmon, indicating that *K. thyrsites* was not evenly distributed throughout fish musculature. Dawson-Coates et al. (2003) investigated the relationship between flesh quality and the numbers of parasite spores and plasmodia in various muscular host sample sites. In the somatic musculature, the mean spore counts from all the tissue sites were the best predictor of *post-mortem* fillet degradation. Similarly, St-Hilaire et al. (1997a) found a significant correlation between post-harvest fillet texture and the number of *K. thyrsites* spores in the somatic muscle tissue of farmed Atlantic salmon, while Funk et al. (2007) revealed that among several analytic methods, SSU rDNA copy number of the parasite was the best predictor of the *post-mortem* flesh quality of *K. thyrsites*-infected farmed salmon. However, the aforementioned studies were based on a few defined muscle tissue samples of infected fish, mostly from the dorsal fillet sections, thus not covering all parts of the fillets examined in our study.

The present findings of highest density in mackerel fillet sections V1 and V2, which again were significantly positively correlated with myoliquefaction score, were clearly influenced by just two (out of 15) host specimens showing highest density and myoliquefaction score (4 and 5, respectively). Thus, based on the present results, we cannot conclude if higher density in the ventral fillet sections reflects a general pattern that governs the development and extent of *K. thyrsites*-induced myoliquefaction in mackerel. Since there was no effect of fish host weight on neither parasite density nor myoliquefaction score, the two smallest mackerel which were also the most densely infected in the sample, could not alone explain the observed correlations in the present sample of *K. thyrsites*-infected mackerel. A similar infection and distribution pattern as observed in our study was also reported for *K. inornata* in spotted seatrout (Ware et al. 2014). The parasite species seems to have an overall nonuniform distribution in the fish host’s somatic musculature, with a trend towards higher plasmodial density in the anterior hypaxial part of the muscle which largely corresponds to the present fillet sections V1 and V2. The authors suggested that the sporadic high density and variation in *K. inornata* plasmodial distribution they observed could in part be due to multiple infections with the parasite over time in some fish, or that varying dosages of actinospores initially infected the fish. Since the life cycle of *K. thyrsites* is still unknown, it is not possible to accurately explain the observed variations in *K. thyrsites* density in the somatic musculature of mackerel. It can, however, not be ruled out that recurrent infections may take place in mackerel, e.g. during spawning migrations, or as juveniles (Levsen et al. 2008). NEA mackerel juveniles live close to the seabed (Jansen et al. 2015) which may facilitate contact with, or at least proximity to the putative alternate host and, hence, the fish infective stage of the parasite.

Assessment of *Kudoa* spp. density based on qPCR seems to be more accurate compared to counting spores or plasmodia using a microscope (Funk et al. 2008), because early stages and unsporulated plasmodia may be difficult to detect and other host cells (e.g., cell nuclei) may resemble *K. thyrsites* unmatured spores*.* Moreover, quantifying infections with this method is extremely time-consuming if accurate density or intensity estimates are sought (Funk et al. 2007). Multiple tissue samples across whole fillets should provide a precise picture of the actual quantitative *Kudoa* infection status in given samples of fish and can reveal sites where the parasite is most prevalent or show highest densities. Representative sites can then be selected for more extensive sampling.

The high sensitivity of qPCR combined with no signs of myoliquefaction has resulted in that most of the apparently intact mackerel sampled as negative controls turned out to be *K. thyrsites*-positive in the fillets and internal organs (see Table 1). In a recent long-term investigation of *K. thyrsites* in NEA mackerel, Giulietti et al. (2022) found that the majority of infected mackerel did not develop ‘soft flesh’ and that only individuals with high parasite density in the somatic musculature showed this condition. Indeed, the present *K. thyrsites*-positive ‘negative control’ mackerel had much lower density in all organs examined, compared to the ‘soft flesh’-affected fish.

Histological examination revealed no evidence for inflammatory host response to the parasite’s plasmodia in the somatic muscle tissue of mackerel, thus confirming the findings of Levsen et al. (2008). Even around ruptured plasmodia and when mature myxospores were dispersed within the tissue, no host reactions were observed. If *intra-vitam* plasmodial rupturing occurs in mackerel, one would expect to find at least some host reactions, e.g. as seen in Atlantic salmon (Moran et al. 1999b) and mahi mahi (*Coryphaena hippurus*) (Langdon 1991). Therefore, *K. thyrsites* in mackerel likely remains hidden within single muscle fibres, while the fish host is still alive. The mechanism behind plasmodial rupturing and spore dispersion has not been clarified. A drop of pH in the musculature after fish death can promote *K. thyrsites*-related proteolytic enzyme activation (Funk et al. 2008). In addition, mechanical influence of the fish during fishing (crowding), pumping, and dense onboard storage may facilitate the rupture of plasmodia and muscle fibres. Even texture testing, done with the present mackerel samples, could have had an effect. This is also in line with observations by Marshall et al. (2015) who found free *K. thyrsites* spores within and between liquefied myocytes of farmed Atlantic salmon. The authors proposed this to be primarily a *post-mortem* effect since loose spores were more frequently observed in fish that were dissected one day post sampling. Thus, the presence of liquefied fish muscle tissue infected with *K. thyrsites* appears to be closely associated with ruptured plasmodia and free myxospores, i.e. intact plasmodia still containing mature myxospores apparently do not induce any myoliquefaction of the surrounding muscle fibres (see also Levsen et al. 2008).

We observed a weak but significantly positive correlation between myoliquefaction score and parasite density, especially in the ventral fillet sections, which was reflected by the histological findings. Low to moderate myoliquefaction (liquefaction scores 2 and 3) was histologically characterized by areas with clearly deteriorated muscle tissue typically showing many aggregated or dispersed spores surrounded by bundles of striated and apparently intact muscle fibres. In sections of score 4 muscle tissue, intact myofibres were largely absent. In sections of score 2 and 3 fillets, myoliquefaction was always accompanied by the presence of free dispersed myxospores. In sections of score 4 fillets on the other hand, intact spores seemed to be absent in strongly deteriorated muscle tissue areas. Instead, scattered deeply stained spots occurred. Since the nuclei of intact muscle fibres of mackerel are typically thin and elongated in shape and were only rarely observed in strongly liquefied muscle tissue, the small spots may be pycnotic nuclei of necrotic cells, possibly including cells from disintegrating myxospores. A weakly stained envelope associated with these spots was sometimes observed, that could be spore valve remains. Transmission electron microscopy (TEM) could be used to determine the origin of these structures.

The association between the dispersion of myxospores within infected fish muscle and the level of muscle myoliquefaction has not been demonstrated for *K. thyrsites* before. Yokoyama et al. (2004) found histologically that the liquefaction of the somatic musculature of Chinese sea bass was associated with the dispersion of free mature myxospores of *K. lateolabracis*. Similarly, Kristmundsson and Freeman (2014) reported that the level of myoliquefaction in Atlantic lumpfish infected with *K. islandica* was dependent upon the number of free myxospores within the affected muscle tissue. However, it is not clear from these reports if the observed dispersion of free spores along with myoliquefaction was induced *post-mortem* or not. Nevertheless, these findings are in line with the present observations and indicate that the distribution of free myxospores and the level of liquefaction of the fish host muscle are interlinked. Since this feature apparently exists in several ‘soft flesh’-inducing *Kudoa* species, the chain of events that eventually leads to focal or generalized myoliquefaction could be advantageous to the parasites. Liquefaction caused by protease released by *K. thyrsites* following death of the host may facilitate spore release to the environment and the perpetuation of the parasite life cycle (Funk et al. 2008).

The somatic musculature appears to be the organ with clearly highest prevalence and density of *K. thyrsites* in mackerel, as well as other fish host species (reviewed by Henning et al. 2013). Plasmodia and myxospores are the microscopically visible stages of the parasite within the muscle tissue. However, DNA traces or cryptic stages of *K. thyrsites* (PCR-based and/or histologically) have also been detected in the blood (Moran et al. 1999b; Marshall et al. 2015) and in the cardiac muscle (Moran et al. 1999b; Young and Jones 2005; Di Cicco et al. 2017) as well as in the gills, skin and intestine (Moran et al. 1999b) of Atlantic salmon. However, that host mounts an immune reaction to the parasite, leading to a degradation of plasmodia that can release spores or spore-containing phagocytes to the blood, being responsible for PCR positive blood and other organs (Marshall et al. 2015). In NEA mackerel, such a host reaction has never been observed. Still, the parasite has been molecularly detected in the blood (Giulietti et al. 2022), as well as in the spleen, kidney, liver, and cardiac muscle, besides somatic muscle (Levsen, 2015; present study). *K. thyrsites* occurred at much lower densities in the internal organs compared to the fillets, which may indicate that the visceral organs do not represent a long-lasting infection site for the parasite in the fish host.

Molecular detections of *K. thyrsites* in blood and internal organs of NEA mackerel and salmon, as well as in the gills of salmon, may have been due to extrasporogonic blood stages that were circulating through these organs (reviewed by Feist et al. 2015; reviewed by Molnár and Eszterbauer, 2015; Moran et al. 1999b). Cryptic stages in the blood may be presporogonic stages, possibly originating from a recent infection (Marshall et al. 2015). Alternatively, there may be traces of parasite DNA in the blood, possibly originating from spores from past infections (Giulietti et al. 2022). It is currently not clarified if recurrent infections of the fish host may occur in *K. thyrsites*. From the present results, no conclusions can be drawn with regard to the temporal aspect of the infection; i.e. if young, comparatively newly established plasmodia do occur, which would be indicative of a rather recent infection. However, from previously examined NEA mackerel samples covering a wider range of fish sizes, Levsen et al. (2008) suggested that the *K. thyrsites* plasmodial development is slow in this fish host species, and that the infection was acquired at an earlier phase of life, e.g. during first time spawning.

Molecular detections of *K. thyrsites* in blood of mackerel (Giulietti et al. 2022) may explain the relatively high occurrence and density of *K. thyrsites* in the heart ventricle observed in this study. Indeed, positive signal in the heart may originate from extrasporogonic blood stages that were circulating through this organ. However, it is also possible that parasite development occurs in the heart as demonstrated by the detection of early developmental stage of *K. thyrsites* in the cardiac muscle of salmon using histology, ISH, and ICH (Moran et al. 1999b; Young and Jones 2005; Di Cicco et al. 2017). The preliminary histological examination of *K. thyrsites* qPCR-positive heart samples of mackerel, however, did not show any *Kudoa*-related stage (data not published). Thus, further investigations should be conducted to clarify if parasite development occurs in the heart of mackerel in the same way as in salmonids.

The *K. thyrsites* tropism towards the somatic musculature and at least in part, the heart, seen here in mackerel substantiates previous observations from other hosts and may be selectively advantageous. Intracellularity in the somatic and in the cardiac musculature hides the parasite from the immune system, allows slow development and greater longevity in the host, and therefore likely increased transmission potential and completion of the life cycle (Pereira et al. 2019). Thus, the proteolytic enzyme production and activity may counteract the ‘hidden’ tissue location of *K. thyrsites* by facilitating the release of the myxospores (Funk et al. 2008).

## Data Availability

All data generated or analysed during this study are included in this published article.

## Declarations

### Animal handling and use

No approval of research ethics committees was required to accomplish the goals of this study because no live animals were used in the experimental work.
